# Enhanced HPV16 E6/E7^+^ tumor eradication via induction of tumor-specific T cells by therapeutic vaccination with virosomes presenting synthetic long peptides

**DOI:** 10.1007/s00262-023-03462-y

**Published:** 2023-05-24

**Authors:** Toon Stegmann, Anna-Sophia Wiekmeijer, Kitty Kwappenberg, Suzanne van Duikeren, Farien Bhoelan, Denzel Bemelman, Thomas J. M. Beenakker, Willem-Jan Krebber, Ramon Arens, Cornelis J. M. Melief

**Affiliations:** 1 Mymetics BV, J.H, Oortweg 21, 2333 CH Leiden, The Netherlands; 2 ISA Pharmaceuticals BV, De Limes 7, 2342 DH Oegstgeest, The Netherlands; 3 Immunology department, Leiden Medical Center, Albinusdreef 2, 2333 ZA Leiden, The Netherlands

**Keywords:** Cancer immunotherapy, Cancer vaccines, Human Papilloma virus, Cervical cancer

## Abstract

Therapeutic cancer vaccines trigger CD4 + and CD8 + T cell responses capable of established tumor eradication. Current platforms include DNA, mRNA and synthetic long peptide (SLP) vaccines, all aiming at robust T cell responses. SLPs linked to the Amplivant® adjuvant (Amplivant-SLP) have shown effective delivery to dendritic cells, resulting in improved immunogenicity in mice. We have now tested virosomes as a delivery vehicle for SLPs. Virosomes are nanoparticles made from influenza virus membranes and have been used as vaccines for a variety of antigens. Amplivant-SLP virosomes induced the expansion of more antigen-specific CD8 + T memory cells in ex vivo experiments with human PBMCs than Amplivant-SLP conjugates alone. The immune response could be further improved by including the adjuvants QS-21 and 3D-PHAD in the virosomal membrane. In these experiments, the SLPs were anchored in the membrane through the hydrophobic Amplivant adjuvant. In a therapeutic mouse model of HPV16 E6/E7^+^ cancer, mice were vaccinated with virosomes loaded with either Amplivant-conjugated SLPs or lipid-coupled SLPs. Vaccination with both types of virosomes significantly improved the control of tumor outgrowth, leading to elimination of the tumors in about half the animals for the best combinations of adjuvants and to their survival beyond 100 days.

## Introduction

Therapeutic cancer vaccines can exert strong anti-cancer activity without serious side effects. Current platforms include DNA, mRNA and synthetic long peptide (SLP) vaccines, all aiming at robust T cell responses [1, 2]. The local and systemic immunosuppression characteristic of late-stage cancer calls for combination therapy with other immunomodulators [1]. Conceivably, the immunogenicity of cancer vaccines can be further enhanced to induce even stronger cytotoxic T cell (CTL) and CD4 + T helper cell responses. The magnitude of SLP vaccine-induced human Papilloma virus type 16 (HPV16)-specific T cell responses correlates with clinical outcome [3, 4]. Further increasing this response might result in even better clinical outcomes. Vaccination with either full-length proteins or peptides presenting the minimal 8–10 amino acid cytotoxic T lymphocyte (CTL) epitope as antigens has proved suboptimal [1, 5]. Synthetic long peptides (SLPs), peptides of 20–39 amino acids in length that require uptake and processing by dendritic cells (DCs) and include both T helper and CTL epitopes, have been shown to induce strong T cell-mediated antitumor responses [1, 5–7]. The addition of Toll-like receptor (TLR) adjuvants to SLPs and the formulation of SLPs in emulsions have been investigated, with CpG ODN 1826 and Montanide ISA 51 VG emulsions containing poly I:C among the most successful ones in mice [8] [9]. Vaccination with SLPs in Montanide has proved to be clinically effective in patients with HPV16 + premalignant or malignant diseases and in patients with melanoma [2].

Recently, SLPs were covalently coupled with adjuvants. Coupling ensures delivery of adjuvant and peptide to the same antigen-presenting cell (APC), thus activating only the APC that receives the peptides and delivering the peptides only to APCs that are activated. SLPs coupled to the TLR-2 adjuvant Pam_3_CSK_4_ [10] or an optimized Pam_3_CSK_4_ derivative, Amplivant®, were demonstrated to be processed by human APCs and induce T helper type 1 responses in an ex vivo model [11]. The same constructs were studied in an in vivo model, in which the Amplivant®-conjugated SLPs were superior in inducing antitumor activity [11]. Amplivant-conjugated peptides could be further improved by introducing a valine–citrulline bond that can be cleaved in the endosome, between adjuvant and peptide [12]. Recently, a clinical study was published in which two SLPs conjugated to Amplivant were used to vaccinate patients with HPV16^+^ (pre)malignant lesions [13]. This study demonstrated that the vaccine was safe and overall well tolerated; furthermore, all patients in the highest dose group displayed a strong HPV16-specific T cell response after vaccination.

APCs take up particles much more efficiently than peptides [14]. Adjuvants can also be associated non-covalently with antigens as part of a complex, or particle, for example, in a complex with albumin [15] to promote uptake, but such complexes can dissociate in vivo. Antigens and adjuvants can also be combined in a virosome. Virosomes are particles of about 100 nm in diameter, produced from the reconstituted membranes of animal viruses, mostly influenza virus [16]. Influenza virosomes retain the hemagglutinin protein (HA) of the virus, which promotes the uptake of the virosomes by APCs and the delivery of antigens to the cytosol by fusion between the endosomal and the virosomal membrane [17, 18]. Virosomes can be produced with peptide or protein antigens covalently linked to lipids of the membrane to induce strong immune responses against those antigens [19–21] and have been marketed as influenza and Hepatitis A vaccines [16, 22]. Virosomes have been shown to induce strong CTL responses [23, 24], and virosomes presenting the HPV16 E7 protein induced the survival of vaccinated mice challenged with TC-1 cells [18].

We have now combined the antigen-presenting power of virosomes with SLP to test their potential as therapeutic cancer vaccines. The SLPs were either linked to Amplivant, with Amplivant both serving as an adjuvant and through its lipid moiety as an anchor for the peptide in the virosomal membrane, or coupled to lipids present in the virosomal membrane via a disulfide bond. In addition, the effect of the additional adjuvants QS-21 and 3D PHAD was tested, which were also incorporated into the virosomal membrane. 3D PHAD is a synthetic version of MPLA, a TLR-4 targeting adjuvant, which has been used in virosome vaccines [25]. QS-21, an inflammasome-activating adjuvant and MPLA are frequently used together in liposomes in marketed vaccines, including prophylactic cancer vaccines [26]. QS-21 is a saponin adjuvant. Although its mechanism of action is not fully understood, it activates the inflammasome and is well known to be particularly effective at inducing CD8^+^ T cells [27, 28]. Our ex vivo experiments with human monocyte-derived dendritic cells (moDCs) show that more antigen-specific CD8 + T cells were stimulated by Amplivant-SLPs presented on virosomes than with Amplivant-SLPs alone. In vivo therapeutic vaccination of virosomes with Amplivant-SLP and lipid-coupled SLP with additional adjuvants led to control of tumor outgrowth and the survival of mice beyond the 100-day duration of the experiment. Virosome incorporation of the adjuvant QS-21 contributed substantially to these activities. These data indicate that SLP-virosomes show great potential as therapeutic cancer vaccines.

## Material and methods

### Preparation of virosomes

Virosomes were prepared under aseptic conditions as described [29]. Briefly, beta-propiolactone-inactivated influenza A/PR/8/34 virus was dissolved for one hour at 25 °C in the short chain phospholipid 1,2-dicaproyl-sn-glycero-3-phosphocholine (DCPC, Avanti Polar Lipids, Birmingham, Alabama) at 100 mM in HNE buffer: 145 mM NaCl, 5 mM HEPES (2-[4-(2-hydroxyethyl)piperazin-1-yl]ethanesulfonic acid), 1 mM EDTA (2,2',2'',2'''-(Ethane-1,2-diyldinitrilo)tetraacetic acid), and then the viral nucleocapsids were removed by ultracentrifugation. The supernatant was mixed with the Amplivant conjugated-SLPs (Amplivant-SLP) or lipidated SLPs dissolved in DMSO (at a 6:1 ratio of HNE to DMSO), and the DCPC and DMSO were removed by dialysis against 7 changes of HNE in a gamma-irradiated Slide-a-Lyzer dialysis cassette (Thermo Fisher Scientific) with a 10-kDa cutoff. Adjuvants were added by post-insertion after the production of virosomes. QS-21 (Desert King, San Diego, CA) was added from stock solution in 10 mM MES, 140 mM NaCl, pH 6.5. 3D-PHAD (Avanti Polar Lipids, Birmingham, AL) was added from a stock solution in DMSO. Incorporation of peptides or adjuvants into the virosomes was investigated by subjecting the virosomes to equilibrium density ultracentrifugation for 64 h at 120.000 g_av_ in a 10–60% sucrose gradient in HNE. The SLP concentration in the fractions was analyzed by SDS-PAGE against an SLP standard, the total phospholipid concentration by phosphate analysis [30], cholesterol by HPLC (Acquity UPLC® BEH C8 1.7 µm column, isocratic elution with methanol/water/trifluoroacetic (TFA) acid 95: 5: 0.1) at 35 ℃, the adjuvant 3-D-PHAD by TLC (a 10 × 10 cm 60 µm silicagel, eluted with chloroform/methanol/water 100: 75: 15 and stained with cerium cesium ammonium molydate), QS-21 by HPLC (Acquity UPLC® BEH C18 1.7 µm column (2.1 × 150 mm) running a gradient of 40–60% B/A (A:water + 0.1%TFA, B: acetonitril + 0.1%TFA)) at 60 ℃) and sucrose concentration by refractometry. The size distribution of the virosomes was measured by single particle tracking sizing analysis on an LM-10 Nanosight® (Malvern, Almelo, Netherlands).

### Peptides

The solid-phase peptide synthesis was performed on a Tetras peptide synthesizer (Advanced ChemTech) by solid-phase Fmoc/tBu chemistry according to established methods [31]. Peptides were conjugated to Amplivant as described [32]. Amplivant was produced by using the chirally pure Fmoc-Cys((R)-2,3-di(palmitoyloxy)-propyl)-OH. Peptides were also coupled to the lipid phosphatidylethanolamine via a disulfide bridge by reacting the cysteine of the peptide close to the C-terminus with 1,2-dioleoyl-sn-glycero-3-phosphatidylethanolamine-N-[3-(2-pyridyldithio)propionate] (PDP-PE, Avanti Polar Lipids), dissolved in 100 mM DCPC/HNE or DMSO with the peptide dissolved in 100 mM DCPC/HNE for 2.5 h at room temperature. The HPV16 E7-derived peptide G2357S (GQAEPDRAHYNIVTFCCKCDS) was used in murine in vivo experiments, and TRPILSPLTKGILGFVFTLTVPSERGLQRRRFV (T3756V), derived from the M1 protein of influenza A/PR/8/34 [33], was used for in vitro experiments with human cells.

### Ex vivo studies with human PBMCs

Buffy coats from anonymous HLA-A2^+^ healthy blood bank donors were obtained after written informed consent (Sanquin, The Netherlands). PBMCs were isolated by Ficoll gradient centrifugation. The PBMCs were cultured in IMDM medium (Lonza, The Netherlands, Belgium) supplemented with 8% human AB serum (BioIVT, UK), 100 U/mL penicillin, 100 µg/mL streptomycin and 2 mM L-glutamine and were stimulated (2*10^6^/cells/well of 24-well plate) for one week with various vaccine preparations. Monocyte-derived DCs (moDCs) were generated by selecting CD14^+^ cells from PBMC using magnetic beads according to the manufacturers’ instructions (Miltenyi Biotec, Leiden, The Netherlands). Isolated cells were plated at 1.2*10^6^/well of a 6-well plate and cultured in the presence of 800 IU/mL GM-CSF and 500 IU/mL IL-4 in IMDM/4% human AB serum for 6 days, after which they were loaded with virosomes or peptide o/n, washed and added to stimulated autologous PBMCs. Samples of the DCs were taken at this point for FACS analysis. After 7 days, PBMCs from the DC/PBMC co-culture were analyzed by flow cytometry. Stimulations were done with 1 µM of SLP or Amplivant-SLP per well.

### Animal experiments

All animal experiments were approved by the ethics committee on animal experimentation of the Leiden University Medical Center. Female C57BL/6 mice were obtained from Janvier Labs and allowed to acclimatize for a week before the start of experiments. Mice were inoculated on day 0 with 1*10^5^ TC-1 cells, randomized for tumor outgrowth on day 8, vaccinated on day 8, a blood sample was taken on day 17, and they were again vaccinated on day 22. Vaccination was done with 20 nmol of SLP, 20 µg CpG ODN1826 (InvivoGen, Toulouse, France), 100 µL Montanide ISA 51 VG (Seppic, France), 3.8 µg of 3D-PHAD or 7.6 µg of QS-21 per mouse per injection. Tumor growth was measured at least twice per week by caliper. Tumor cells and vaccinations were administered subcutaneously in the flanks.

### Flow cytometry

Human PBMC samples were incubated for 30 min at room temperature with an HLA-A2 tetramer containing the GILGFVFTL epitope and thereafter washed and incubated with CD3, CD4 and CD8 antibodies (all antibodies used were from Miltenyi Biotec, Leiden, The Netherlands) for 15 min at 4 °C. Mouse white blood cells were incubated for 30 min at room temperature with an H-2^b^ tetramer containing the RAHYNIVTF epitope and CD3, CD8, CD43 and CD62L-KLRG1 antibodies. Likewise, human DC samples were stained with antibodies for CCR7, CD80, CD86, CD40, CD11c and HLA-DR. After washing, cells were analyzed on a LSR-II (BD Biosciences) at the Flow Cytometry Facility of the LUMC. Results were analyzed using FlowJo software.

## Results

### Production of virosomes containing Amplivant-linked peptides

**Fig. 1:**
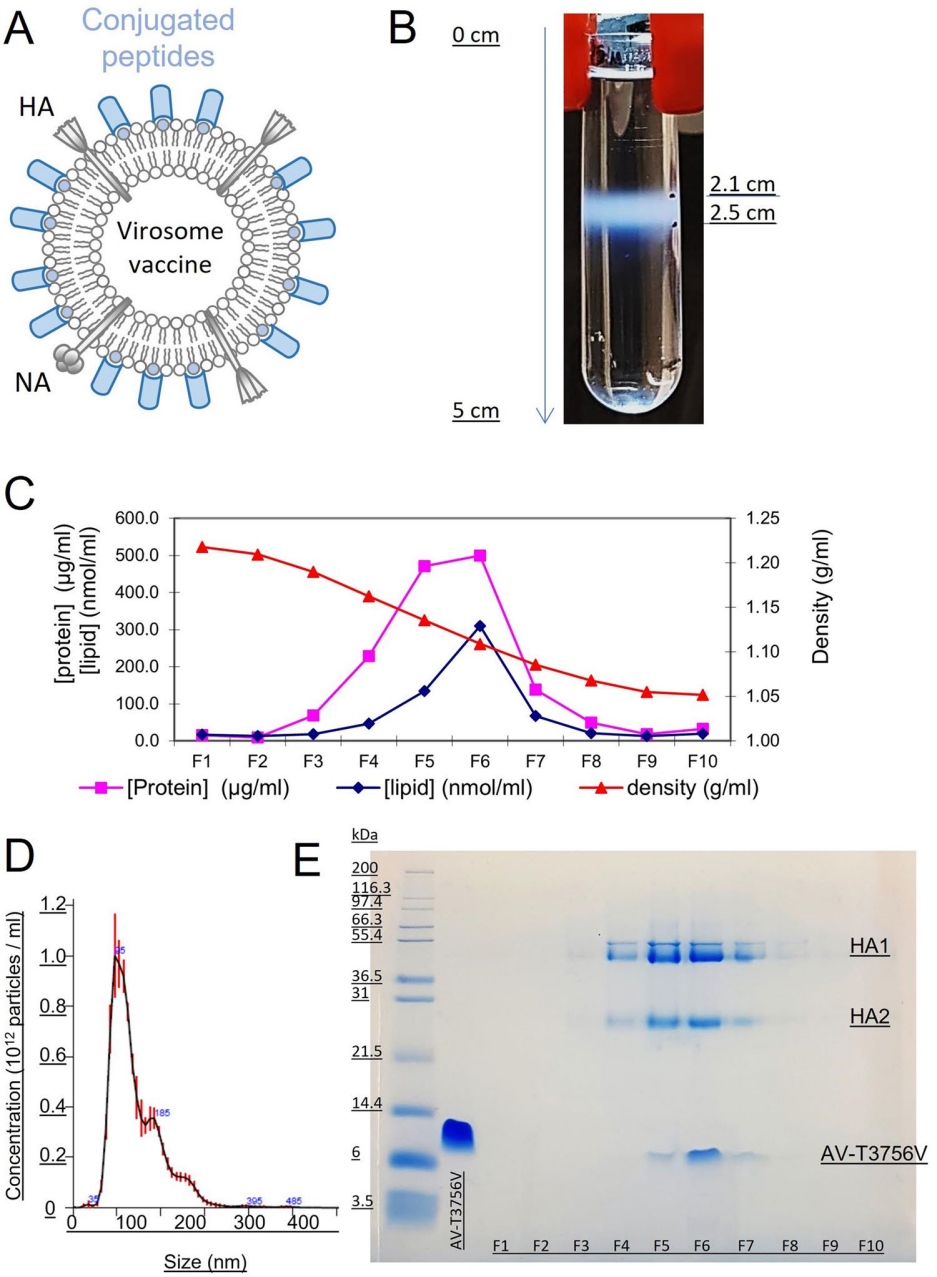
Equilibrium density sucrose gradient analysis of virosomes. Schematic diagram of virosomes, showing the viral membrane lipids, viral proteins HA and NA, and the Amplivant-, or lipid conjugated peptide **A**. Virosomes were spun at 120.000 g for 64 h in a 10–60% sucrose gradient, showing a single band **B** with a density of around 1.1 g/ml. Fractions **C** were taken from bottom (left) to top and assayed for protein, lipid (phosphate) and sucrose concentration (converted to density) and by SDS-PAGE gel **E**, AV-T3756V is the Amplivant-conjugated SLP (left, fractions to the right), HA1 and HA2 are the subunits of influenza hemagglutinin. The size distribution of the virosomes **D** was measured by single particle tracking sizing analysis (Nanosight®)

Virosomes were produced by mixing DCPC-solubilized viral membranes, containing the lipids and the HA protein of influenza virus, with Amplivant-SLPs in DMSO, and then, the DCPC and DMSO were removed by dialysis, resulting in reconstitution of the viral membranes, now including the amphiphilic Amplivant-SLP. Equilibrium density centrifugation on sucrose gradients was then used to analyze incorporation of Amplivant-SLP in the virosomes, and size analysis of the virosomes was done by single particle tracking analysis (Fig. 1). The virosomes migrated as a single band on the gradient, containing all the viral lipids, the HA protein and the Amplivant-SLP. Amplivant alone, without the coupled SLP, was similarly incorporated in the viral membrane (not shown). These data indicate that Amplivant-SLP molecules were firmly integrated into the virosomal membrane, most likely with the hydrophobic part of the adjuvant residing in the interior of the membrane bilayer. The virosomes had a modal size of 82 nm and the size distribution showed a peak with a shoulder toward larger diameters, possibly indicating aggregation or the presence of some larger particles.

### Ex vivo induction of memory T cell responses

To test the immunostimulant effect of virosomes containing Amplivant-SLPs, loaded human moDCs were cocultured with autologous stimulated peripheral blood monocytes (PBMCs). The T3756V SLP, containing the influenza M1 epitope GILGFVFTLTV[33], was used. SLP, Amplivant-SLP and Amplivant-SLP incorporated into virosomes with or without adjuvant(s), and virosomes without antigen and adjuvant(s) were tested. The studied additional adjuvants were QS-21 and 3D-PHAD alone or in combination at different concentrations. Adjuvants were incorporated into the virosomal membrane by post-insertion and their incorporation determined as described above.

Upon stimulation of DCs with different vaccine preparations, Amplivant-SLP virosomes lowered the viability of DCs by around 30% (Table 1). Virosomes induced the expression of CD11c^+^, CD40 and CD86. Virosomes containing Amplivant-SLP, with or without additional adjuvant(s), strongly stimulated the expression of CCR7 on DCs, a marker for migration of the cells toward lymph nodes, more so than SLP only, Amplivant-SLP or virosomes without antigen (Table 1). No additional stimulatory effect of QS-21 or 3D-PHAD on CCR7 expression was seen in comparison with Amplivant-SLP virosomes; in fact, at intermediate concentrations of QS-21, the % CCR-7 high cells was lower.

**Table 1:** moDC markers 1 day after stimulation. The geometric mean expression relative to no treatment (“none”’) was expressed as the fold change for CD11c, CD40, CD86 and CCR7 gated on live cells and % CCR7 + high moDCs gated on CD11c + /CD40 + . H,M,L: concentrations of virosome-included 3D-PHAD 0.95 µg/ml (H) 0.19 µg/ml (M) 0.038 µg/ml (L), and QS-21 H: 0.95 µg/ml, M, 0.38 µg/ml, L: 0.076 µg/ml

Vaccine			Cells		Relative geometric mean expression of marker (fold change)			
	QS21	3D PHAD	% in live gate	% CCR7 high	CD11c	CD40	CD86	CCR-7
None			71.9	1.08	1.00	1.00	1.00	1.00
Peptide alone			75.6	0.95	0.99	0.97	0.88	1.01
Amplivant-SLP			70.0	1.52	0.83	0.99	1.39	1.16
Empty virosomes			77.2	1.16	1.37	1.17	1.24	1.40
Amplivant-SLP virosomes			51.2	3.49	1.37	1.27	1.70	1.64
Amplivant-SLP virosomes	High		48.3	2.91	1.17	1.23	1.80	1.58
Amplivant-SLP virosomes	High	High	57.3	3.63	1.31	1.39	2.06	2.42
Amplivant-SLP virosomes	High	Medium	51.9	3.76	1.45	1.48	2.13	2.01
Amplivant-SLP virosomes	High	Low	48.8	3.29	0.83	0.94	1.80	2.41
Amplivant-SLP virosomes	Medium		50.5	2.01	1.43	1.35	1.97	1.61
Amplivant-SLP virosomes	Medium	High	53.6	1.82	0.82	0.96	1.58	2.25
Amplivant-SLP virosomes	Medium	Medium	53.5	2.49	1.04	1.36	2.18	1.08
Amplivant-SLP virosomes	Medium	Low	61.5	1.85	1.19	1.23	1.82	1.85
Amplivant-SLP virosomes	Low		50.2	3.18	1.28	1.15	1.80	1.63
Amplivant-SLP virosomes	Low	High	60.1	2.47	1.28	1.40	2.08	2.48
Amplivant-SLP virosomes	Low	Medium	60.7	2.18	0.67	0.86	1.68	1.74
Amplivant-SLP virosomes	Low	Low	55.0	2.86	1.10	1.27	2.12	1.17
Amplivant-SLP virosomes		High	43.1	3.41	1.29	1.29	1.80	1.64
Amplivant-SLP virosomes		Medium	48.9	3.55	1.41	1.40	1.97	2.31
Amplivant-SLP virosomes		Low	56.9	4.09	1.34	1.37	1.96	2.73

**Fig. 2:**
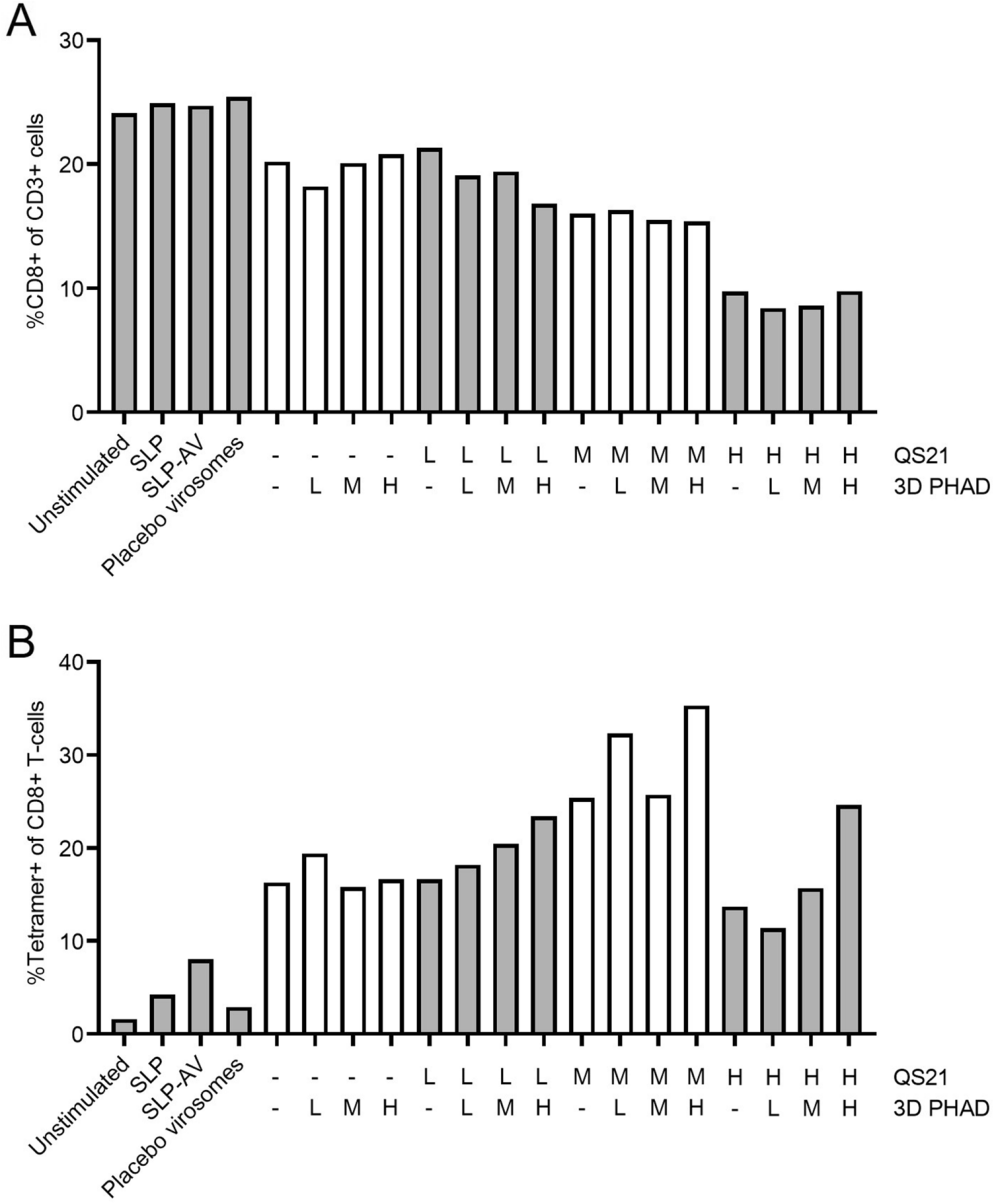
Expansion of human influenza-specific T cells in vitro after stimulation of autologous DC with different virosome SLP vaccine formulations. **A** % CD8 + T cells (of live CD3 + cells), **B** Tetramer positive CD8 T cells (of live CD3 + cells). Preparations tested were: buffer only (unstimulated), peptide alone (SLP), Amplivant-SLP (SLP-AV), virosomes without antigen (Placebo virosomes), virosomes containing Amplivant; H,M,L: concentrations of virosome-included 3D-PHAD 0.95 µg/ml (H) 0.19 µg/ml (M) 0.038 µg/ml (L), and QS-21 H: 0.95 µg/ml, M, 0.38 µg/ml, L: 0.076 µg/ml. SLP concentration was 1 nmol/ml

Upon restimulation of PBMCs with the DCs, the percentage of CD8 + T cells among CD3 + cells was lower when virosomes containing QS-21 had been added to the cultures, while addition of 3D-PHAD had no effect (Fig. 2A). There was no change in CD4 + T cells as a percentage of CD3 + T cells; the peptide does not contain a CD4 epitope. The effect of QS-21 on the frequency of CD8 + T cells was dose dependent. Stimulation with SLP induced a slight increase in the percentage of antigen-specific CD8 + T cells as measured by tetramer staining (Fig. 2B). Conjugation of SLP to Amplivant further increased the frequency of tetramer-positive cells (7.5%). Inclusion of Amplivant-SLP in virosomes doubled that percentage (16%), and the inclusion of the adjuvants QS-21 and 3D-PHAD at the optimum concentration (1 µM of Amplivant-SLP plus 0.38 mg/ml of QS-21 and 0.95 µg/ml 3 D PHAD) more than doubled that percentage again, to 35% (Fig. 2B). The immunostimulatory effect of QS-21 displayed a clear optimum, being highest at an intermediate concentration (0.38 µg/ml) 3D-PHAD alone, or QS-21 alone at the low (0.076 µg/ml) or high (0.95 µg/ml) concentrations tested had only a limited stimulating effect. Addition of 3D-PHAD to QS-21 had a clear additional effect at the highest and lowest QS-21 concentration tested, but a more modest additional effect at the optimum concentration of QS-21.

Thus, the highest concentration of QS-21 had a negative effect on the viability of CD8^+^ T cells, but induced more antigen-specific CD8^+^ T cells. However, the intermediate concentration of QS-21 induced the highest percentage of antigen-specific T cells that was increased further by the addition of 3D PHAD.

**Fig. 3:**
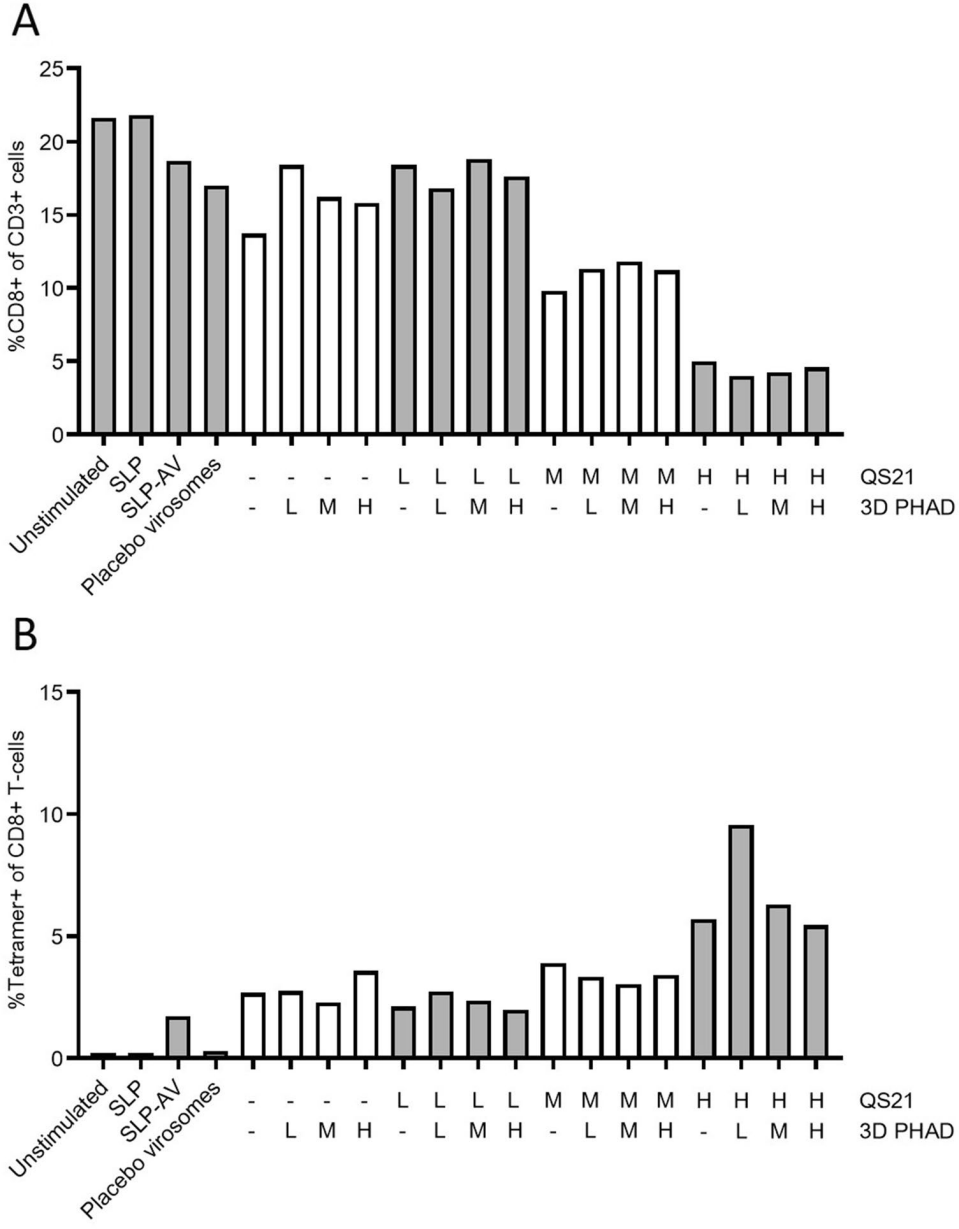
Second donor: **A** % CD8 + T cells (of live CD3 + cells), **B** Tetramer positive CD8 T cells (of live CD3 + cells). Preparations tested were: buffer only (unstimulated), peptide alone (SLP), Amplivant-SLP (SLP-AV), virosomes without antigen (Placebo virosomes), virosomes containing Amplivant-SLP (H,M,L: concentrations of virosome-included 3D-PHAD 0.95 µg/ml (H) 0.19 µg/ml (M) 0.038 µg/ml (L), and QS-21 H: 0.95 µg/ml, M, 0.38 µg/ml, L: 0.076 µg/ml, SLPs concentration 1 nmol/ml in all cases

Cells from a second donor generated a lower overall immune response (Fig. 3), most likely due to a lower precursor frequency of Influenza M1-specific CD8 + T cells, as observed in the unstimulated (neg) sample, from 1.7% tetramer-specific CD8 + T cells for stimulation with the Amplivant-SLP to 2.7% for virosomes containing Amplivant-SLP to 9.6% for virosomes additionally containing the optimal concentrations of QS-21 and 3D-PHAD (0.95 µg/ml QS-21 QS-21 plus 0.038 µg/ml 3D PHAD). Overall, these results confirmed the observations made with cells from the first donor. In conclusion, virosomes with Amplivant-SLP activate moDCs, to induce tetramer-positive T cells, and the immune response can be enhanced by including the adjuvants QS-21 and 3D PHAD in the virosomal membrane. Because of the differences between donors, a precise optimum concentration for QS-21 cannot be determined, but it is in the medium to high range.

### In vivo* experiments*

The above results indicated that the CD8^+^ T cell response to an Amplivant-SLP conjugate can be augmented by presenting this conjugate on a virosome and can be further increased by including a combination of the adjuvants QS-21 and 3D-PHAD. To study the efficacy of virosomes containing Amplivant-SLP in a therapeutic vaccination setting, the HPV16 E6/E7^+^ TC-1 tumor model was used. Mice were inoculated with tumor cells to create established tumors and were vaccinated twice with virosomes, Amplivant-SLP alone or SLP emulsified with Montanide ISA 51 VG and CpG ODN 1826.

Amplivant has a dual role in the virosome-based vaccines; it serves as an adjuvant, but at the same time as an anchor for the peptide in the membrane of the virosomes. To better understand the contribution of these two functions, lipid-SLPs were prepared, where instead of Amplivant, a phospholipid present in all cell membranes, phosphatidylethanolamine, without adjuvant properties, serves as the membrane anchor. The phospholipid was linked via a disulfide bridge to the C-terminal cysteine of the peptide, and the conjugate incorporated into virosomes similar as for Amplivant-SLP.

**Fig. 4:**
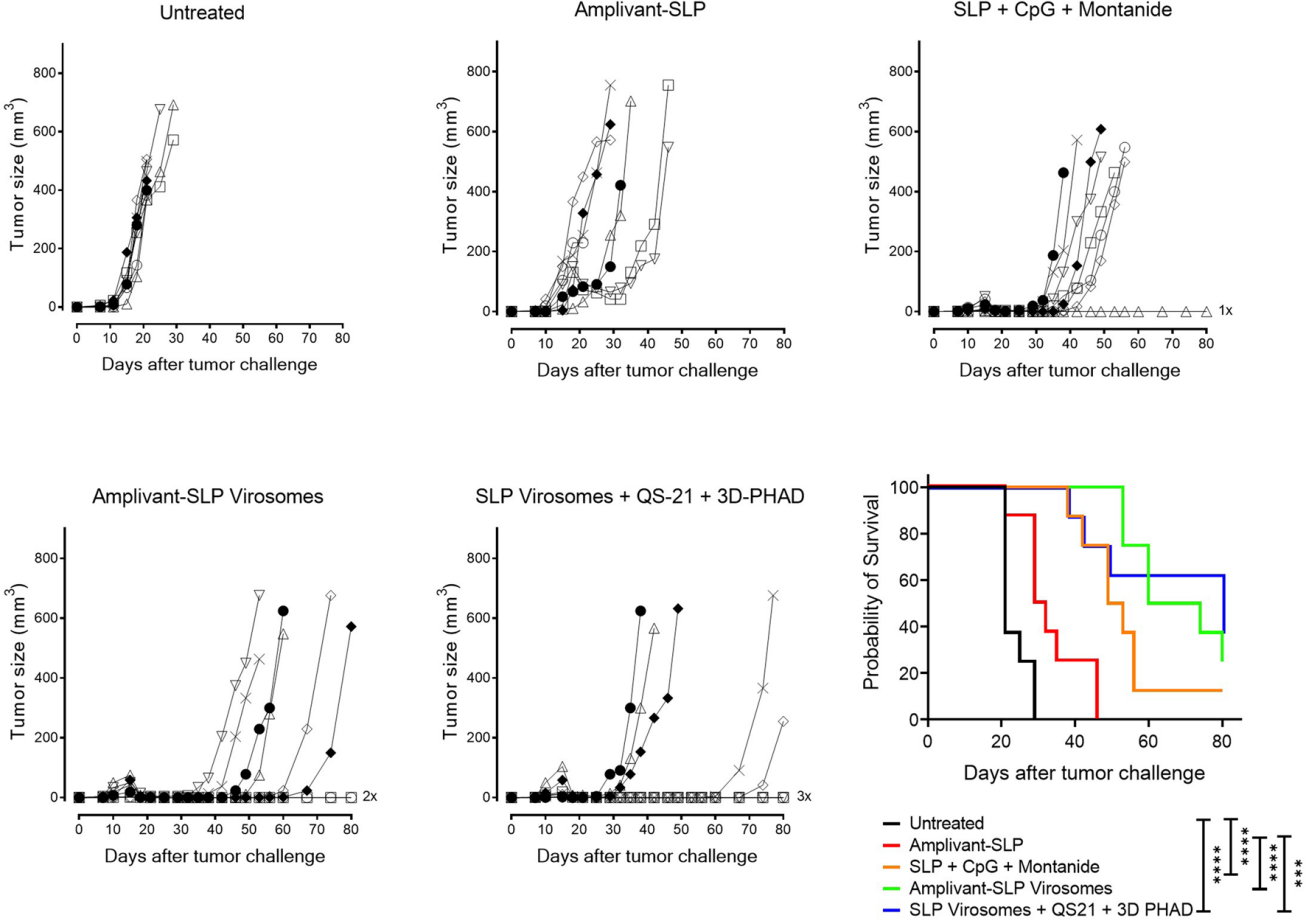
Outgrowth of TC-1 tumor and survival of C57BL/6 mice after vaccination with different virosome SLP formulations. Tumor outgrowth in mice (*n* = 8/group) inoculated with TC-1 cells on day 0, randomized for tumor growth and vaccinated on day 8, and again vaccinated on day 22, using the vaccine preparations indicated. Mice were killed when the tumors reached 500 mm^3^ or after 80 days. Kaplan–Meier graphs of survival: Significant differences (log rank test): both virosome groups vs. untreated animals (*p* < 0.0001) and Amplivant-SLP virosomes vs. Amplivant-SLP: *P* < 0.0001, lipid-SLP virosomes plus adjuvants vs. Amplivant-SLP: *p* = 0.0011. Other comparisons: Amplivant-SLP virosomes vs. lipid-conjugated virosomes with QS-21 and 3D-PHAD: *p* = 0.7435 Amplivant-SLP virosomes vs. SLP + Montanide conjugated SLP: *p* = 0.057 Lipid-conjugated virosomes with adjuvants vs. SLP + CpG + Montanide: *p* = 0.2042. *p < 0.05, **p < 0.01, ***p < 0.001, ****p < 0.0001

Compared to unvaccinated mice, Amplivant-SLP alone delayed tumor outgrowth (Fig. 4) and significantly improved overall survival (*p* < 0.01) (Fig. 4), while SLP adjuvanted with Montanide and CpG delayed tumor growth, improving survival. Virosomes presenting Amplivant-SLP as well as virosomes with SLP coupled to phospholipid plus QS-21 and 3D-PHAD adjuvant both clearly controlled TC-1 tumor outgrowth in the mice, which resulted in significantly improved survival compared to untreated animals (*p* < 0.001, Fig. 4), leading to 2/8 and 3/8 mice surviving beyond the 80 days of the experiment, respectively. For both these virosome preparations, the differences with Amplivant-SLP not present in virosomes were also significant (*p* < 0.001 for Amplivant-SLP virosomes vs. Amplivant SLP, and *p* = 0.0011 for lipid-SLP virosomes vs. Amplivant SLP). Virosomes with lipid-anchored SLP are thus at least as suitable as Amplivant-SLP containing virosomes for therapeutic vaccination in the TC-1 model, implying that lipid-coupled SLPs are correctly processed for antigen presentation.

**Fig. 5:**
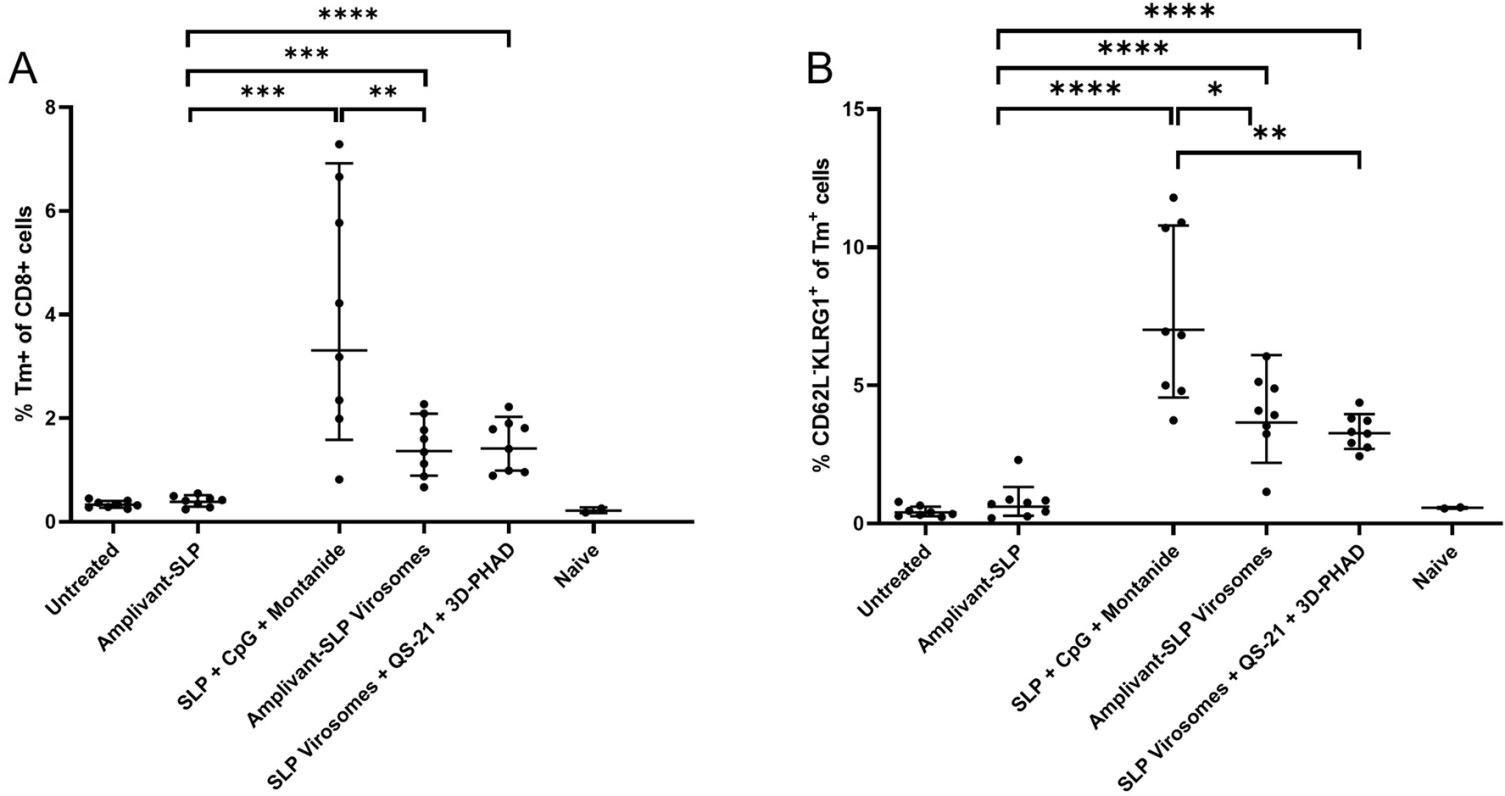
Analysis of peripheral T cells harvested from mice upon termination. **A** Tetramer-positive T cells (gated on live CD3 + , CD8 +) and **B** effector memory T cells (CD62L-, KLRG1 + cells gated within the tetramer-positive cells). *p < 0.05, **p < 0.01, ***p < 0.001

Both virosome vaccine formulations induced an increase in peripheral tetramer-positive CD8 + T cells and effector memory (CD62L-KLRG-1 +) CD8 + T cells. However, there was no clear correlation with survival or control of tumor outgrowth as higher levels of these populations were induced in mice vaccinated with SLP emulsified in Montanide with CpG (Fig. 5).

**Fig. 6:**
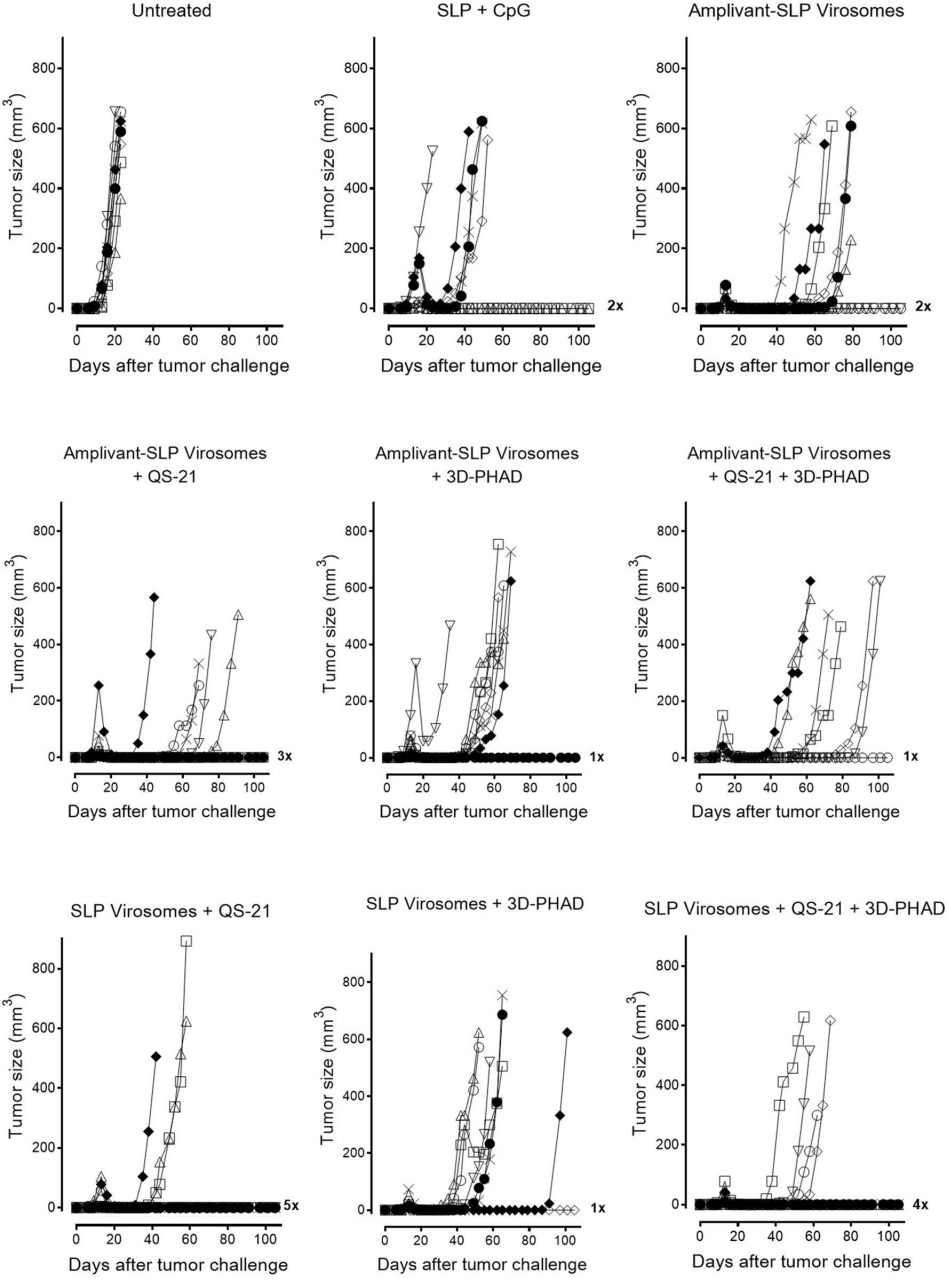
Outgrowth of TC-1 tumor and survival of C57BL/6 mice after vaccination with different virosome formulations. Groups of 8 mice each were inoculated with TC-1 cells on day 0, randomized for tumor growth and vaccinated on day 8, and again vaccinated on day 22, using the vaccine preparations indicated. Mice were killed when the tumors reached 500 mm^3^ or after 100 days

**Fig. 7:**
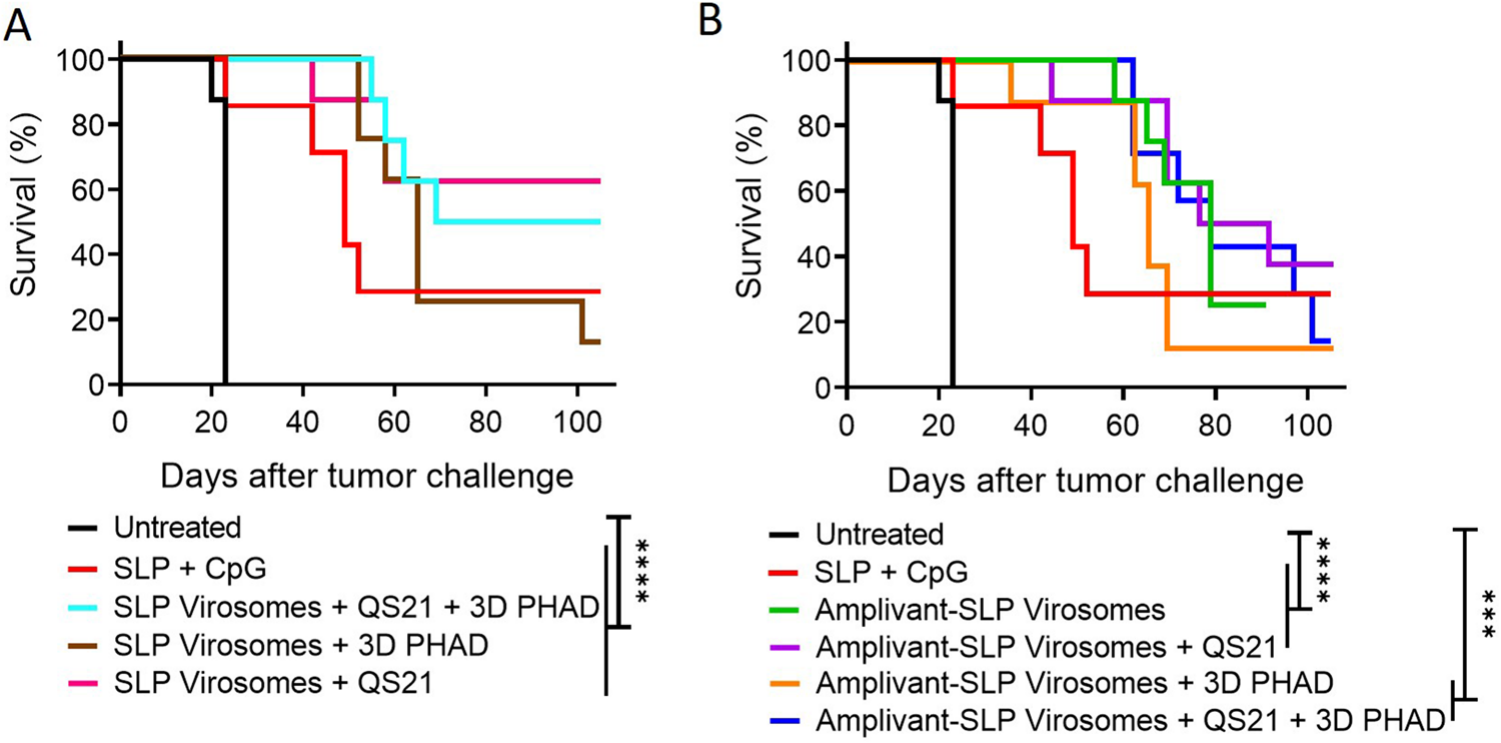
Kaplan–Meier graphs of the survival of mice shown in Fig. 6. **A** Amplivant-SLP virosomes vs. untreated mice and mice vaccinated with SLP and CpG. **B** Lipid-linked SLP virosomes vs. the same controls. Significant differences (log rank test): all virosome groups vs. untreated animals (*p* < 0.0001, except Amplivant-SLP virosomes + 3D PHAD *p* = 0.0001 and Amplivant-SLP virosomes + QS-21 + 3D-PHAD *p* = 0.0003). The differences between virosome groups were not significant, and the differences between virosome groups and SLP + CpG and Montanide were also not significant. *p < 0.05, **p < 0.01, ***p < 0.001, ****p < 0.0001

These experiments demonstrate that virosomes with Amplivant-SLP and also virosomes with SLP coupled to lipid are suitable for use in therapeutic cancer vaccines, the latter if they also contain the adjuvants QS-21 and 3D-PHAD. We therefore tried to combine these adjuvants also with Amplivant-SLP virosomes and investigated the contribution of each of these adjuvants to lipid-SLP virosomes individually (Figs. 6 and 7). It was again found that virosomes with Amplivant-SLP controlled tumor outgrowth associated with 25% (2/8) of mice surviving beyond the 100 days of the experiment. Adding QS-21 to Amplivant-SLP virosomes delayed tumor outgrowth even more, but did not significantly improve survival, while the addition of 3D-PHAD, both alone and in combination with QS-21, seemed to have a negative effect on survival compared to virosomes with Amplivant-SLP alone. Virosomes containing lipid-linked peptide plus QS-21 alone were very effective in controlling tumor outgrowth, leading to the survival of 62.5% (5/8) mice for > 100 days (Fig. 7). 3D-PHAD on its own was less effective as an adjuvant, and in combination with QS-21, it was even less effective than QS-21 alone. If the results between lipid-coupled SLP and Amplivant-SLP were compared for each adjuvant, the differences were not significant (QS-21, *P* = 0.53, 3D-PHAD *P* = 0.62, QS-21 plus 3D-PHAD *p* = 0.47).

**Fig. 8:**
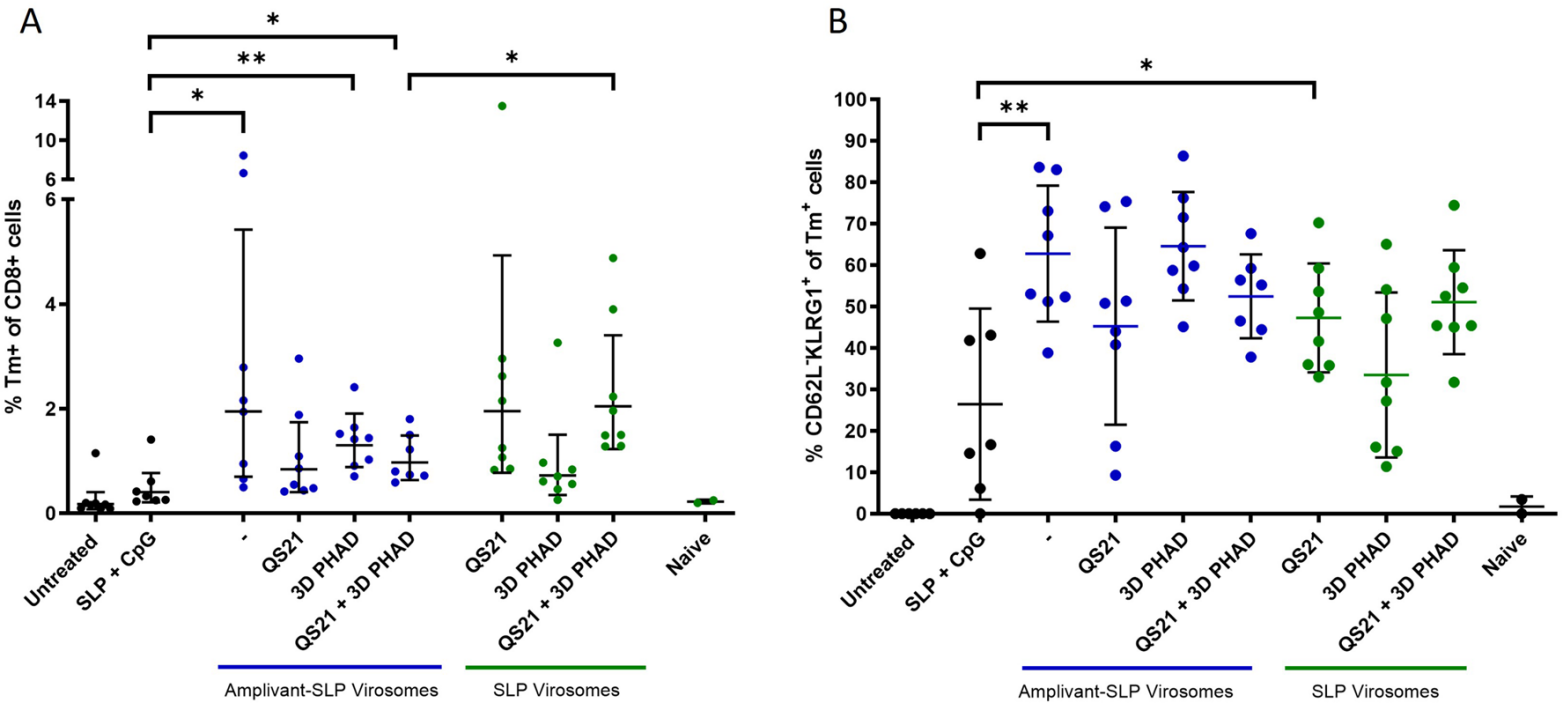
Analysis of peripheral T cells harvested from mice upon termination. **A** tetramer-positive T cells (on live CD3 + , CD8 + , gate). **B** Effector memory T cells (CD62L-, KLRG1 + cells gated on the tetramer-positive cells). None of the differences were significant

Both types of virosomes induced an increase in peripheral tetramer-positive CD8 + T cells and effector memory (CD62L-KLRG-1 +) CD8 + T cells, but there was no clear correlation with survival or tumor outgrowth data (Fig. 8).

## Discussion

Therapeutic cancer vaccines aim to induce robust T cell responses targeted at tumor antigens. Such responses can be enhanced by combining the optimal antigen with adjuvants and by presenting the antigen on particles. Antigen-presenting cells take up particles much more efficiently than proteins [14], and preferably, antigen and adjuvant are taken up by the same cell to ensure both efficient co-stimulation and antigen presentation. In order to arrive at an optimal vaccine platform, we have used the following important elements in our strategy: (1) SLPs were used as antigens; SLPs, in contrast with short peptides, can only be processed and cross-presented efficiently by professional antigen-presenting cells, thus preventing presentation by non-professional cells, which can lead to tolerization [34]. Promising clinical results have already been obtained with therapeutic vaccination using SLPs targeting HPV16 E6 and E7 oncoproteins combined with chemotherapy in cervical cancer [4] [2] and combined with anti-PD-1 in head & neck cancer [35, 36], using Montanide as an adjuvant (2) Modification of the peptides with Amplivant fosters induction of Th1 T helper responses and sustained and robust CTL responses [37]. (3) Virosomes are nanoparticles that are particularly effective at presenting a variety of antigens, from HIV proteins and peptides to allergy-related antigens [19, 21]. Virosomes without adjuvant containing soluble HPV 16 E7 protein in the lumen of the virosomes can induce CD8 + T cells and prevent tumor outgrowth in a prophylactic vaccination model [38]. Virosomes, when added as an adjuvant to soluble peptides can provide T cell help for CTL induction [39]. Also, ovalbumin coupled to virosomes can induce CD4 + immune responses [40]. When we coupled SLPs to the membrane of virosomes, their incubation with DC clearly allowed efficient MHC-I processing and presentation. (4) Finally, we have included additional hydrophobic adjuvants, such as QS-21 and 3D-PHAD, in the membrane of the virosomes, trying to further enhance the immune response.

In vitro, in a human PBMC model system with an influenza matrix SLP, Amplivant-SLP in virosomes, when used to stimulate human moDCs, expanded greater numbers of influenza-specific CD8 + memory T cells in PBMCs than Amplivant-SLP alone, and the presence of the additional adjuvants QS-21 and 3D-PHAD in the virosomal membrane further enhanced this antigen-specific expansion. Thus, when using human primary cells, there appears to be synergy between different elements of the virosomal vaccine formulation, and the SLPs are correctly delivered and processed.

In therapeutic cancer vaccine experiments in mice, Amplivant-SLP in virosomes led to a significantly better survival than Amplivant-SLP alone. This result and those of the in vitro experiments were somewhat surprising given that the SLP was anchored in the membrane by the Amplivant adjuvant through Amplivant’s hydrophobic lipid tail. Amplivant is a TLR-2 adjuvant; TLR-2 is a receptor present on the cell membrane of myeloid cells, including professional APCs like DCs. Thus, the adjuvant might have become inaccessible to the receptor by the membrane integration; on the other hand, it has been demonstrated that TLR-2 ligand linked peptides were taken up into APCs independent of cell surface TLR-2 expression and still stimulated the immune response [41]. We therefore also tested the effect of anchoring of the SLP in the virosomal membrane by coupling a free cysteine in the SLP to a household phospholipid that does not function as an adjuvant, via a disulfide bridge, and included a combination of QS-21 and 3D-PHAD as adjuvant. Surprisingly, this combination worked as well as Amplivant-SLP virosomes for controlling tumor outgrowth and survival, and antigen processing by peripheral T cells. It is therefore possible that the Amplivant does not need to act as an adjuvant when it is embedded in virosomes; the adjuvant effect could instead be provided by the virosomal presentation of SLPs, with the Amplivant functioning as an anchor. To test this hypothesis, in a second set of experiments, Amplivant-SLP virosomes were provided with the same adjuvants as lipid-SLP virosomes, QS-21 and 3D-PHAD, alone or in combination. Indeed, in some of these combinations lipid-SLPs were at least as good as Amplivant-SLPs in terms of survival (Fig. 7). However, the interaction between the adjuvants is complex. 3D-PHAD, a TLR-4 adjuvant, comparable to TLR-2 in its effect, did not provide any benefits to Amplivant-SLP virosomes and actually seemed to decrease the effect of QS-21 on outgrowth and survival for both types of virosomes. In conclusion, it seems possible that Amplivant primarily served as a lipid anchor in these experiments.

Lipid-coupled SLP in virosomes with QS-21 adjuvant seemed to provide the best therapeutic effect (5/8 mice surviving), statistically on a par with Amplivant-SLP virosomes plus QS-21. The presence of a disulfide bridge between lipid and peptide may also have been beneficial; in the reducing environment of the cytosol where antigen processing for MHC class I exposition takes place, the peptide may be liberated from the lipid, facilitating processing. Likewise, Amplivant-SLPs conjugated by a valine–citrulline bond between SLP and adjuvant that can be cleaved in the endosome improved the immune response to the SLP [12].

In conclusion, these experiments show that CD8 + immune responses from SLPs can be strongly enhanced by presenting the SLPs coupled to virosomes, using human cells in vitro*,* and virosomes presenting SLPs containing QS-21 adjuvant provide the best control of tumor outgrowth and improved survival in a mouse model.
